# Metabolomic Pathways to Osteoporosis in Middle‐Aged Women: A Genome‐Metabolome‐Wide Mendelian Randomization Study

**DOI:** 10.1002/jbmr.3358

**Published:** 2018-01-26

**Authors:** Alireza Moayyeri, Ching‐Lung Cheung, Kathryn CB Tan, John A Morris, Agustin Cerani, Robert P Mohney, J Brent Richards, Christopher Hammond, Tim D Spector, Cristina Menni

**Affiliations:** ^1^ Department of Twin Research & Genetic Epidemiology King's College London London UK; ^2^ Farr Institute of Health Informatics Research Institute of Health Informatics University College London London UK; ^3^ State Key Lab of Pharmaceutical Biotechnology Hong Kong China; ^4^ Department of Pharmacology and Pharmacy University of Hong Kong Pokfulam Hong Kong China; ^5^ Centre for Genomic Sciences University of Hong Kong Pokfulam Hong Kong China; ^6^ Department of Medicine University of Hong Kong Pokfulam Hong Kong China; ^7^ Department of Human Genetics McGill University Montreal Canada; ^8^ Centre for Clinical Epidemiology, Lady Davis Institute, Jewish General Hospital McGill University Montreal Canada; ^9^ Department of Epidemiology Biostatistics, and Occupational Health McGill University Montreal Canada; ^10^ Metabolon, Inc. Durham NC USA; ^11^ Department of Medicine McGill University Montreal Canada

**Keywords:** METABOLOMICS, GENOMEWIDE ASSOCIATION STUDIES, MENDELIAN RANDOMIZATION, INSTRUMENTAL VARIABLES ANALYSIS, BONE MINERAL DENSITY, OSTEOPOROSIS

## Abstract

The metabolic state of the body can be a major determinant of bone health. We used a Mendelian randomization approach to identify metabolites causally associated with bone mass to better understand the biological mechanisms of osteoporosis. We tested bone phenotypes (femoral neck, total hip, and lumbar spine bone mineral density [BMD]) for association with 280 fasting blood metabolites in 6055 women from TwinsUK cohort with genomewide genotyping scans. Causal associations between metabolites and bone phenotypes were further assessed in a bidirectional Mendelian randomization study using genetic markers/scores as instrumental variables. Significant associations were replicated in 624 participants from the Hong Kong Osteoporosis Study (HKOS). Fifteen metabolites showed direct associations with bone phenotypes after adjusting for covariates and multiple testing. Using genetic instruments, four of these metabolites were found to be causally associated with hip or spine BMD. These included androsterone sulfate, epiandrosterone sulfate, 5alpha‐androstan‐3beta17beta‐diol disulfate (encoded by *CYP3A5*), and 4‐androsten‐3beta17beta‐diol disulfate (encoded by *SULT2A1*). In the HKOS population, all four metabolites showed significant associations with hip and spine BMD in the expected directions. No causal reverse association between BMD and any of the metabolites were found. In the first metabolome‐genomewide Mendelian randomization study of human bone mineral density, we identified four novel biomarkers causally associated with BMD. Our findings reveal novel biological pathways involved in the pathogenesis of osteoporosis. © 2017 American Society for Bone and Mineral Research.

## Introduction

It is now established that genetic factors, environmental factors, and their interactions play a major role in osteoporosis pathogenesis.^1^ In addition, metabolic pathways play an important role in age‐related bone loss. Several circulating proteins and metabolites are known biomarkers of bone turnover and can help to identify the state of bone formation/resorption for diagnostic or therapeutic purposes.^2^ Observation of bone loss accompanying several endocrinological, inflammatory, gastrointestinal, renal, and nutritional disorders,^3^ often known as secondary osteoporosis, also suggests that the metabolic state of the body can be a major determinant of bone health. Identification of metabolites that are causally associated with reduced bone mass will increase our power to understand the biological mechanisms of osteoporosis and to develop drug targets for bone health.

Causality, however, cannot be inferred from observational studies,^4^ and randomized controlled trials are considered the gold standard for establishing causal relationships. Recently, Mendelian randomization (MR), an instrumental variable analysis method, has been proposed as an alternative for establishing causal relationships.^5^ For inference of causal relationships between an exposure (eg, a metabolite) and an outcome (eg, bone mineral density [BMD]), a susceptibility variant (or the genetic risk score) of the exposure is used as instrument. The association of this instrument with the exposure is not affected by any confounding factors (because the genetic sequence is randomly assigned during conception), and it is associated with the outcome only via its effects on the exposure.^6^ MR has been successful in inferring causality for several clinically important traits^7^ and for elucidating the complex etiology of common disease.^8^

In this study, we used MR on a genome‐metabolome‐wide scale. We first looked for circulating metabolites associated with bone density measures in a large cohort of twins with metabolomic data. We then searched for the genetic markers of those metabolites associated with BMD and used them as instrumental variables to identify metabolites causally associated with bone health. For evaluating bidirectional effects, we also used genetic risk scores for BMD measures as instrumental variables. Lastly, we replicated our findings in an independent cohort from Hong Kong.

## Materials and Methods

**Figure 1:**
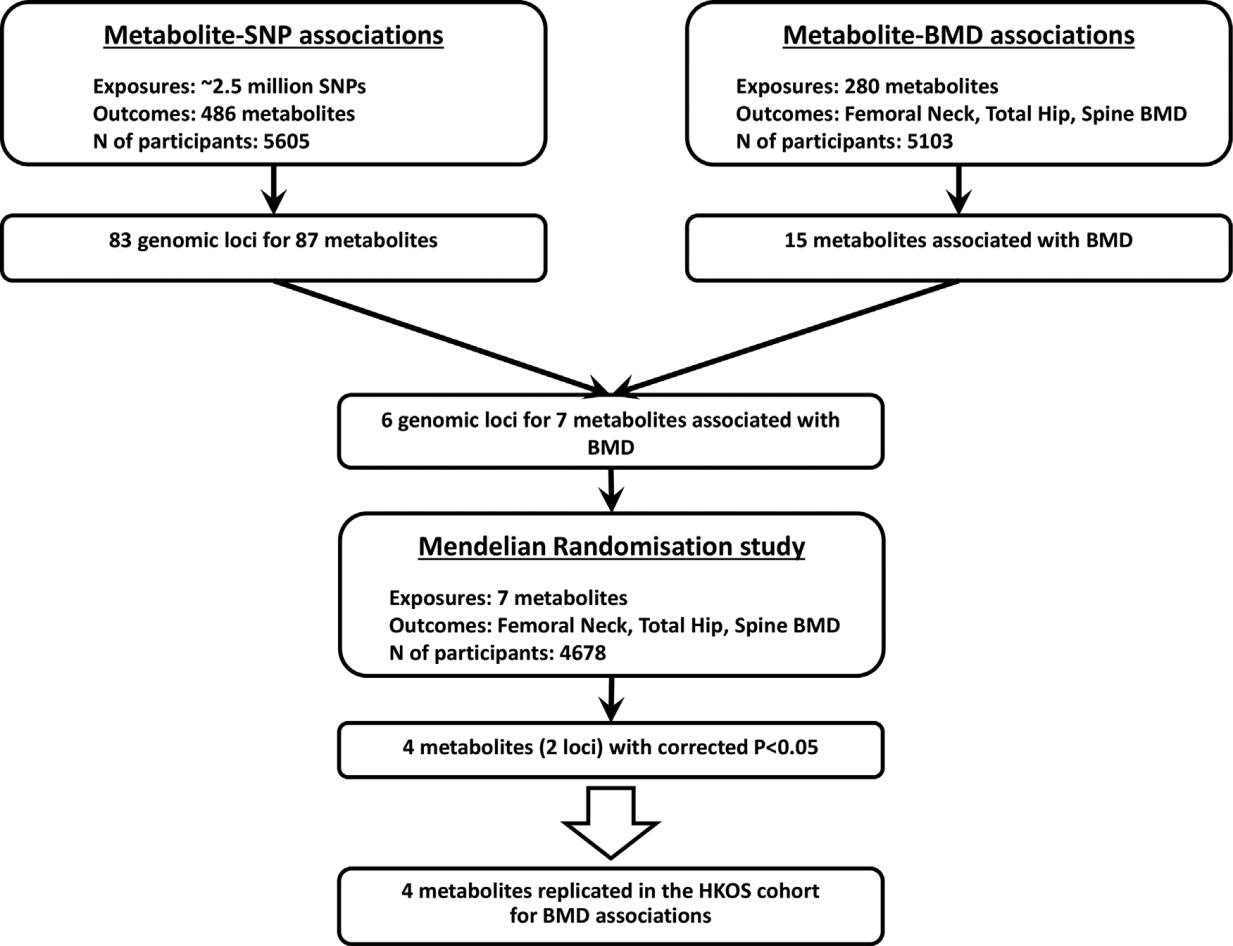
Flow chart of the study.

The flowchart of the study design is presented in Fig. 1.

### Discovery cohort

The TwinsUK study, started in 1992, is a nationwide registry of volunteer twins in the UK, with about 12,000 registered twins (83% female predominantly middle‐aged and older).^9^ The cohort has extensive demographic, physiological, behavioral, and lifestyle data available and is one of the most deeply phenotyped and genotyped cohorts in the world. The study has been an active partner of many large collaborative projects including Genetic Factors for Osteoporosis (GEFOS) consortium.^10^ The study has been approved by St. Thomas’ Hospital Research Ethics Committee, and all twins have provided informed written consent.

Dual‐energy X‐ray absorptiometry (DXA) was used to measure BMD at the lumbar spine (L_1_ to L_4_) and hip regions (Hologic Discovery W devices, Hologic, Bedford, MA, USA). In total, 15,491 hip and spine DXA scans were performed in 7056 twins during 17 years of follow‐up.^11^ All BMD measurements were performed by trained technicians using a standardized protocol of measurement. Daily quality‐control scans were performed using the spine phantom. For this study, we included all female twins with metabolomic data and at least one BMD measurement in hip/spine regions. DXA scans from the same date of blood sampling for metabolomic analysis were used in 4650 twins, and BMD measurements from the closest date (if multiple measurements) were selected for 453 twins without DXA in the same date of blood sampling (up to 2 years differences were allowed). This resulted in a study population of 5103 participants (mean age 53.8 ± 13.8 years).

### Replication cohort

The Hong Kong Osteoporosis Study (HKOS) is a prospective longitudinal cohort study of osteoporosis and fracture that started in 1995. The cohort participants were community‐dwelling Southern Chinese men and women of Han descent recruited from public road shows and health fairs held in various districts of Hong Kong from 1995 to 2010.^12^ A total of 9449 participants were recruited. In 2015, a full‐scale in‐person follow‐up study was conducted. DXA has been used to measure BMD at the lumbar spine (L_1_ to L_4_) and hip regions (Hologic QDR 2000plus and 4500plus systems at baseline; Hologic Discovery A and Horizon A at follow‐up study). All BMD measurements were performed by trained technicians using a standardized protocol. Daily quality‐control scans were performed using the spine phantom and across the DXA machines to make sure of consistent measurements.

### Metabolomic profiling

Non‐targeted metabolite detection and quantification was conducted by the metabolomics provider (Metabolon, Inc., Durham, NC, USA) on fasting blood samples of 6055 twins, as previously described.^13^ This platform incorporates two separate ultra‐high‐performance liquid chromatography/tandem mass spectrometry injections and one gas chromatography/mass spectrometry injection per sample, and can detect metabolites in the range of low nanograms per milliliter.^14^ In previous studies, the platform findings have been shown to be highly stable^15^ and reproducible (with median relative standard deviation of 5% for internal standards added to samples before mass spectrometry).^14^ The same metabolomic profiling was also performed on 340 and 291 serum samples from different participants in the HKOS baseline and follow‐up studies, respectively. Blood samples were taken in the same day of DXA scans for all patients.

In this study, we analyzed 280 structurally named biochemicals (known metabolites) categorized into the following broad categories: amino acids, lipids, carbohydrates, vitamins, nucleotides, peptides, xenobiotics, and steroids.

### Genomewide association study (GWAS)

Genomewide genotyping was performed for 5710 participants of the TwinsUK study using two chips (HumanHap300 BeadChip and HumanHap610 QuadChip, Illumina, San Diego, CA, USA). The genotype data have been imputed (using HapMap II reference panel containing ∼2.5 million autosomal single‐nucleotide polymorphisms [SNPs]) and used in several international consortia for different phenotypes.^10^ In this study, we performed genomewide association studies (GWAS) for all metabolites including those significantly associated with bone phenotypes.^16^ The software MERLIN was used to account for twin structure. Results from the largest GWAS meta‐analysis for BMD measures^17^ were used to calculate genetic risk scores (GRS) for hip and spine BMD.

### Statistical analysis

All models were built using Stata (version 14, StataCorp LLC, College Station, TX, USA) and SIMCA software (version 13, Umetrics, Umeå, Sweden). For quality control of metabolomic data, we had to consider that mass spectrometry was performed over several days for the cohort samples, and measured concentrations were variable according to run days. We, therefore, normalized the metabolite data by dividing each metabolite concentration by its median in the respective run day and then inverse normalized the data because the metabolite concentrations were not normally distributed. Moreover, to avoid spurious false‐positive associations due to small sample size, we excluded metabolomic traits with more than 20% missing values. We imputed individual metabolites not detected in a sample using the minimum measures in their run day. The same QC procedure was performed in HKOS.

#### Association analysis in TwinsUK

To assess the predictive power of combined metabolites, we used orthogonal‐partial least squares (O‐PLS) regression. We used a sevenfold cross‐validation technique to validate the O‐PLS models.^18^ Goodness‐of‐fit measures were reported for all models in training (coefficient of variation labeled as R^2^) and cross‐validation (coefficient of variation labeled as Q^2^) sets. For univariate metabolite‐BMD trait associations, we used mixed‐effects random‐intercept models considering family relatedness and zygosity as random effects variables, and age, height, weight, and duration of hormone‐replacement therapy (HRT; as a continuous variable in units of years) as confounders.

#### Mendelian randomization analysis

For the metabolomic GWAS analysis, we only considered metabolites passing the Bonferroni corrected threshold of *p* < 1.79 × 10^−4^ (0.05 divided by 280, for the number of known metabolites) for association with at least one of the BMD phenotypes. Genomic loci passing a stringent threshold for association with these metabolites (*p* < 1 × 10^−10^ for the sentinel SNP in a locus) as well as hip and spine GRS scores were considered for the bidirectional instrumental variable analysis.^16^ In the direct metabolite‐to‐BMD analysis, metabolite levels were used as endogenous variables; age, height, weight, and duration of HRT were included as exogenous variables; and allele frequency (additive model) was used as the instrument. Bidirectional Mendelian randomization (MR)^19^, ^20^ also investigates for any evidence of reverse causality (ie, BMD causally influencing metabolite levels) by use of GRS scores as the instrument and BMD measures as the endogenous variables for metabolite level outcomes. We used the generalized method of moments (GMM) with cluster‐robust heteroskedastic‐consistent variance estimates.^21^ Under‐, weak‐, and over‐identification limitations were checked. Bootstrap resampling of the data (10,000 repetitions) was used for the calculation of robust estimates of standard errors from instrumental variables analysis.

#### Replication in the HKOS

Multivariable robust regression models were used to evaluate metabolites found to be causally associated with BMD in the TwinsUK cohort. We reported all the association between these metabolites and BMD, adjusting for age, sex, height, and weight from the same visit as DXA and metabolic measurements. HRT users were excluded in the metabolomic study. Analyses were conducted separately in the baseline and follow‐up studies and then meta‐analyzed using inverse variance method.

## Results

The demographic characteristics of the study populations are presented in Table 1.

**Table 1:** Demographic Characteristics of the Study Populations (Mean [SD])

	TwinsUK	HKOS
No.	6055	624
Male/female	434/5621	112/512
Age (years)	53.45 (14.00)	52.05 (14.25)
Body mass index (kg/m^2^)	26.07 (4.91)	22.83 (3.84)
Height (m)	1.62 (0.07)	1.58 (0.08)
Weight (kg)	69.24 (13.78)	56.98 (11.26)
Femoral neck BMD (mg/cm^2^)	786.98 (129.42)	710.62 (167.51)
Total hip BMD (mg/cm^2^)	926.12 (133.67)	815.12 (176.04)
Lumbar spine BMD (mg/cm^2^)	985.58 (152.75)	919.03 (203.01)

SD = standard deviation; HKOS = Hong Kong Osteoporosis Study; BMD = bone mineral density.

### Metabolomic signature of osteoporosis

**Figure 2:**
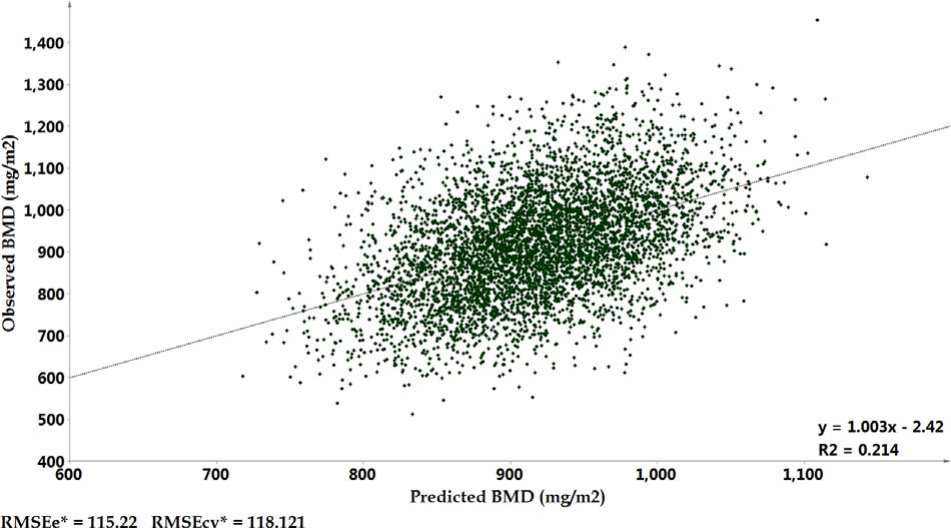
Scatter plot of observed versus predicted total hip bone mineral density (BMD) values based on orthogonally filtered partial least square (O‐PLS) model with 280 metabolites among 4937 participants. *RMSEe = root mean square error of the estimation (the fit) for training sample; RMSEcv = root mean square error for cross‐validation sample.

Principal components analysis (PCA) showed 38 significant components in our panel with the cumulative *R^2^* of 55% in the training set and 35.5% in the cross‐validation set (*Q^2^*). O‐PLS modeling confirmed one component as predictive and two as orthogonal for prediction of BMD in different regions. Models showed higher predictive power of metabolites for total hip BMD (*R^2^* = 21.5% and *Q^2^* = 17.7%) and femoral neck BMD (*R^2^* = 18.9% and *Q^2^* = 15.6%) compared with lumbar spine (*R^2^* = 15.0% and *Q^2^* = 10.5%). A scatter plot of observed versus predicted values for total hip BMD based on the O‐PLS model is presented in Fig. 2.

### Metabolite associations

Applying stringent multiple testing correction for metabolite‐BMD associations, three metabolites were associated with femoral neck BMD, 13 metabolites with total hip BMD, and 10 metabolites with lumbar spine BMD (15 metabolites in total; Table 2). Two metabolites (prolyl‐hydroxyproline and caffeine) showed significant associations with all skeletal sites measured. Several sulfated adrenal androgens (including androsterone, epiandrosterone, dehydroisoandrosterone, 5alpha‐androstan‐3beta,17beta‐diol, and two metabolomic features related to 4‐androsten‐3beta,17beta‐diol) were associated with total hip or spine BMD. Three metabolites related to the metabolic pathways of valine, leucine, and isoleucine metabolism (4‐methyl‐2‐oxopentanoate, alpha‐hydroxyisovalerate, and 3‐methyl‐2‐oxovalerate) and one related to tryptophan metabolism (C‐glycosyltryptophan) were also associated with BMD.

**Table 2:** Metabolites Significantly Associated With BMD Phenotypes in TwinsUK Population

Trait	Metabolite	Beta (SE)	*p* Value
Femoral neck BMD	Prolyl‐hydroxyproline	–11.0 (1.75)	3.8 × 10^−10^
	Caffeine	6.8 (1.66)	3.8 × 10^−5^
	C‐glycosyltryptophan	–7.1 (1.85)	1.2 × 10^−4^
Total hip BMD	Prolyl‐hydroxyproline	–14.57 (1.76)	2.3 × 10^−16^
	4‐androsten‐3beta,17beta‐diol disulfate 1	13.48 (2.07)	9.6 × 10^−11^
	Dehydroisoandrosterone sulfate (DHEAS)	12.39 (2.02)	1.0 × 10^−9^
	4‐methyl‐2‐oxopentanoate	9.79 (1.79)	4.9 × 10^−8^
	C‐glycosyltryptophan	–9.8 (1.87)	1.8 × 10^−7^
	Epiandrosterone sulfate	9.4 (1.85)	3.9 × 10^−7^
	Androsterone sulfate	8.94 (1.85)	1.4 × 10^−6^
	4‐androsten‐3beta,17beta‐diol disulfate 2	10.37 (2.18)	2.1 × 10^−6^
	5alpha‐androstan‐3beta,17beta‐diol disulfate	9.27 (2.07)	8.0 × 10^−6^
	Caffeine	7.21 (1.75)	3.8 × 10^−5^
	Pipecolate	6.81 (1.68)	5.1 × 10^−5^
	Alpha‐hydroxyisovalerate	6.99 (1.77)	8.2 × 10^−5^
	3‐methyl‐2‐oxovalerate	6.98 (1.8)	1.0 × 10^−4^
Lumbar spine BMD	Prolyl‐hydroxyproline	–16.97 (2.2)	2.0 × 10^−14^
	4‐androsten‐3beta,17beta‐diol disulfate 1	15.36 (2.61)	4.6 × 10^−9^
	Creatinine	10.25 (2.21)	3.6 × 10^−6^
	5alpha‐androstan‐3beta,17beta‐diol disulfate	11.87 (2.56)	3.8 × 10^−6^
	5alpha‐pregnan‐3beta,20alpha‐diol disulfate	11.38 (2.56)	9.5 × 10^−6^
	Dehydroisoandrosterone sulfate (DHEAS)	11.02 (2.49)	9.9 × 10^−6^
	Alpha‐hydroxyisovalerate	9.24 (2.22)	3.2 × 10^−5^
	Pipecolate	8.62 (2.16)	6.6 × 10^−5^
	Caffeine	8.39 (2.16)	1.0 × 10^−4^
	4‐methyl‐2‐oxopentanoate	8.4 (2.18)	1.2 × 10^−4^

BMD = bone mineral density; SE = standard error.

### Mendelian randomization

Results of the bidirectional MR analyses for all metabolites are shown in the Supplemental Material. In the direct instrumental variables analysis, four sulfated adrenal androgen levels (androsterone sulfate, epiandrosterone sulfate, 5alpha‐androstan‐3beta,17beta‐diol disulfate, and 4‐androsten‐3beta,17beta‐diol disulfate 1) showed causal associations with BMD traits (Table 3). We could not see any evidence for a reverse relationship (ie, BMD causing any metabolite level changes). The first three metabolites were instrumented on SNP rs4646450 (chromosome 7q22.1), an intron variant of the *CYP3A5* gene (cytochrome P450, family 3, subfamily A, polypeptide 5). This gene encodes a member of the cytochrome P450 superfamily of enzymes. These enzymes catalyze many reactions involved in drug metabolism and synthesis of cholesterol, steroids (eg, testosterone, progesterone, and androstenedione), and other lipids. The other metabolite was instrumented on SNP rs16981893 (chromosome 19q13.33). This SNP is downstream of the *SULT2A1* gene (sulfotransferase family, cytosolic, 2A, dehydroepiandrosterone‐preferring, member 1). Sulfotransferases aid in the metabolism and excretion of drugs and endogenous compounds. The product of *SULT2A1* catalyzes the sulfation of steroids and bile acids in the liver and adrenal glands.

**Table 3:** Metabolites Showing a Significant Causal Association to Various BMD Traits in Mendelian Randomization Study in TwinsUK Population

							Assoc. (SNP ∼ Met)		Assoc. (BMD ∼ Met)		Assoc. (SNP ∼ BMD)		IV (SNP → Metabolite → BMD)		
Metabolite	SNP	Chr	Locus name	EA/OA	N	Trait	Beta (SE)	*p* Value	Beta (SE)	*p* Value	Beta (SE)	*p* Value	Beta (SE)	*p* Value	95% CI (10,000 permutations)
Androsterone sulfate	rs4646450	7q22.1	CYP3A5	G/A	4638	FN‐BMD	–0.393 (0.035)	7.7 × 10^–29^	6.11 (1.93)	1.6E–03	–10.23 (3.5)	0.0035	25.9 (9.0)	0.0041	(8.2, 43.6)
						TH‐BMD	–0.393 (0.035)	7.7 × 10^–29^	9.15 (1.96)	3.1E–06	–10.25 (3.63)	0.0048	25.9 (9.2)	0.0049	(7.9, 44.0)
						SP‐BMD	–0.393 (0.035)	7.7 × 10^–29^	6.98 (2.47)	4.8E–03	–11.4 (4.58)	0.0129	29.1 (11.9)	0.0147	(5.7, 52.6)
															
Epiandrosterone sulfate	rs4646450	7q22.1	CYP3A5	G/A	4638	FN‐BMD	–0.345 (0.034)	3.1 × 10^–23^	6.55 (1.94)	7.3E–04	–10.23 (3.5)	0.0035	29.6 (10.5)	0.0047	(9.1, 50.1)
						TH‐BMD	–0.345 (0.034)	3.1 × 10^–23^	9.59 (1.98)	1.4E–06	–10.25 (3.63)	0.0048	29.6 (11)	0.0073	(7.9, 51.3)
						SP‐BMD	–0.345 (0.034)	3.1 × 10^–23^	7.22 (2.45)	3.3E–03	–11.4 (4.58)	0.0129	33.3 (13.5)	0.0140	(6.7, 59.8)
															
5alpha‐androstan‐3beta,17beta‐diol disulfate	rs4646450	7q22.1	CYP3A5	G/A	3363	FN‐BMD	–0.211 (0.04)	9.9 × 10^–8^	5.92 (2.2)	7.1E–03	–10.23 (3.5)	0.0035	62.8 (22.8)	0.0059	(18.1, 107.5)
						TH‐BMD	–0.211 (0.04)	9.9 × 10^–8^	9.7 (2.24)	1.5E–05	–10.25 (3.63)	0.0048	56.6 (23.8)	0.0172	(10.0, 103.1)
						SP‐BMD	–0.211 (0.04)	9.9 × 10^–8^	12.96 (2.73)	2.2E–06	–11.4 (4.58)	0.0129	64.2 (28.7)	0.0256	(7.8, 120.5)
															
4‐androsten‐3beta,17beta‐diol disulfate 1	rs16981893	19q13.33	SULT2A1	G/A	3242	FN‐BMD	0.157 (0.032)	7.2 × 10^–7^	9.2 (2.21)	3.2E–05	5.72 (3.34)	0.0870	59.7 (28)	0.0329	(4.8, 114.5)
						TH‐BMD	0.157 (0.032)	7.2 × 10^–7^	14.47 (2.29)	3.2E–10	8.33 (3.5)	0.0176	71.6 (31)	0.0207	(10.9, 132.2)
						SP‐BMD	0.157 (0.032)	7.2 × 10^–7^	16.46 (2.83)	7.0E–09	15.66 (4.34)	0.0003	110.4 (37)	0.0032	(37.0, 183.7)

BMD = bone mineral density; SNP = single‐nucleotide polymorphism; EA = effect allele; OA = other allele; SE = standard error; IV = instrumental variables; CI = confidence interval; FN‐BMD = femoral neck BMD; TH‐BMD = total hip BMD; SP‐BMD = lumbar spine BMD.

### Replication in the HKOS

To further evaluate the generalizability of these findings, we replicated these causally associated metabolites in the HKOS. All four metabolites were significantly replicated in the HKOS (Table 4). The variance explained (adjusted *R^2^*) by these metabolites were 2.7% and 1.2% for BMD at the total hip and lumbar spine, respectively, after adjustment for age, sex, height, and weight. The corresponding numbers for the TwinsUK population were 0.83% and 0.80%, respectively.

**Table 4:** Replication of Metabolite‐BMD Associations in the Chinese Population for Causally‐Associated Metabolites From TwinsUK Population

		TwinsUK			Chinese cohort		
Trait	Metabolite	*n*	Beta (SE)	*p* Value	*n*	Beta (SE)	*p* Value
Femoral neck BMD	4‐androsten‐3beta,17beta‐diol disulfate 1	4003	7.40 (2.01)	0.000224	624	9.82 (5.59)	0.0788
	Epiandrosterone sulfate	4937	6.89 (1.80)	0.000132	624	20.15 (6.24)	0.0012
	Androsterone sulfate	4937	6.39 (1.81)	0.000425	624	18.11 (7.31)	0.0132
	5alpha‐androstan‐3beta,17beta‐diol disulfate	3675	5.58 (1.99)	0.005133	624	7.42 (2.04)	0.0003
Total hip BMD	4‐androsten‐3beta,17beta‐diol disulfate 1	4003	13.19 (2.05)	1.49 × 10^−10^	624	17.26 (5.93)	0.0036
	Epiandrosterone sulfate	4937	9.67 (1.84)	1.64 × 10^−7^	624	23.45 (6.63)	0.0004
	Androsterone sulfate	4937	9.37 (1.85)	4.21 × 10^−7^	624	23.22 (7.73)	0.0027
	5alpha‐androstan‐3beta,17beta‐diol disulfate	3675	9.36 (2.05)	5.57 × 10^−6^	624	7.77 (2.16)	0.0003
Lumbar spine BMD	4‐androsten‐3beta,17beta‐diol disulfate 1	4003	14.32 (2.59)	3.54 × 10^−8^	624	20.34 (7.78)	0.0089
	Epiandrosterone sulfate	4937	6.69 (2.29)	0.003556	624	27.30 (8.76)	0.0018
	Androsterone sulfate	4937	6.61 (2.32)	0.004363	624	27.59 (10.16)	0.0066
	5alpha‐androstan‐3beta,17beta‐diol disulfate	3675	11.63 (2.54)	5.12 × 10^−6^	624	8.25 (2.80)	0.0032

BMD = bone mineral density; SE = standard error.

## Discussion

To the best of our knowledge, this is the first study that uses two levels of “omics” data to detect the causal associations between blood‐circulating metabolites and BMD variation. We confirmed the causal role of adrenal‐secreted sulfated steroids in osteoporosis and identified two novel genetic loci (*CYP3A5* and *SULT2A1*) to be important in the regulation of metabolites causing osteoporosis. Successful replication of the causally associated metabolites in another ethnic group also demonstrated a high generalizability of our findings.

The contribution of metabolites into BMD variation is not yet established. Previous metabolomic studies have mainly focused on low BMD associations using low‐resolution techniques instead of looking into BMD variations across the whole range in large population cohorts.^22^, ^23^ Using a high‐throughput metabolomic platform measuring 280 known metabolites in a large population, we showed that circulating metabolites explained about 35% to 55% of BMD variation in all skeletal sites measured. This can have important clinical applications because circulating metabolite levels can be used as markers of bone health, predictors of risk of osteoporotic fractures, and markers of treatment effects or potential intervention targets (diet and medications).

Using the MR approach, we found that sulfated adrenal androgen plays a causal role in bone metabolism. These metabolites are related to androgen metabolism, and androgen has long been known as an anabolic agent in bone metabolism. However, whether the elevated levels of these metabolites represent the higher levels of endogenous androgen in the participants or contribute individually to bone metabolism is unknown and needs further assessment. Our previous study showed that one of these metabolites, epiandrosterone sulfate, was significantly associated with chronic widespread musculoskeletal pain.^24^ The SNP rs4646450 has shown significant association with femoral neck BMD in the GEFOS consortium meta‐analysis.^17^ Importantly, all the causally associated metabolites in the TwinsUK study showed direct association with BMD in the HKOS population, and collectively explained 2.7% variance of BMD variation at the total hip. This raises hope for potential feasible metabolic interventions, either diagnostic or therapeutic, in clinical management of osteoporosis.

It is important to note that we cannot exclude a potential causal association of the metabolites associated with BMD in our study but not included in the MR analysis. This is mainly related to the lack of reliable genetic instruments for running MR analyses for these metabolites. For instance, prolyl‐hydroxyproline showed significant association with BMD in all three measurement sites, but we could not find any genetic variant for it in our GWAS. This metabolite is a known marker of bone collagen degradation, and its urinary excretion has been previously linked to postmenopausal osteoporosis.^2^, ^25^ Caffeine also showed direct associations with BMD variation in our study, but this should be interpreted cautiously because the levels of this xenobiotic may only reflect short‐term exposure to coffee in participants and not be related to the risk of osteoporosis associated with long‐term coffee intake. The effect of caffeine on bone has been controversial throughout literature.^26^

Our study shows that combining two sources of “omics” studies (genomics and metabolomics) would be beneficial in uncovering the pathogenesis of osteoporosis and other complex health conditions. Although genetic factors are well‐known markers of osteoporosis, combining 63 susceptibility variants in the largest genomewide meta‐analysis only explained 5.8% of the variance of femoral neck BMD variation.^17^ Although these genetic studies can bring several important clinical applications,^1^ the genetic variations per se are not modifiable and so the findings cannot be directly translated into clinical practice. Using these genetic markers as instruments for discovery of causally associated metabolites can reveal more insight on the pathological pathways to diseases and potential interventions.

Our study has several strengths. This is the first study that used two levels of “omics” data to detect the causal associations between blood‐circulating metabolites and BMD variation. The results were further replicated in another ethnic group, highlighting the high reliability and generalizability of the findings. Both TwinsUK and HKOS are well‐characterized cohorts for osteoporosis. The metabolomic profiling in TwinsUK and HKOS were performed on the same platform by the same provider with a highly stringent quality control, making the metabolomic data comparable between cohorts.

Our study has some limitations. There is a potential for horizontal pleiotropy in our study (ie, variants used as instruments can directly influence other metabolites, which then influence BMD). We could not employ new extensions of the MR method (namely multivariable MR^27^ and MR Egger regression)^28^ because of power limitations. Metabolites were measured in a relative manner and so their absolute concentrations cannot be inferred. Moreover, the difference in levels of metabolites between British and Chinese participants could not be assessed (given separate normalized values in each population). Patients receiving HRT were excluded from the metabolomics and genomics studies in the HKOS cohort at the design stage. This may cause some differences in the target populations of the cohorts, although use of HRT is not common in the Chinese population. False‐negative causal associations are quite likely attributable to lack of validated genetic instruments for several metabolites. Future collaborative studies with larger sample sizes are required to evaluate the role of other BMD‐associated metabolites in bone metabolism.

Our findings have important clinical implications. Currently, there is a crisis in the treatment of osteoporosis worldwide.^29^ Patients are generally afraid of initiating or using anti‐osteoporosis treatments because of the fear of severe but rare side effects. Thus, the development of novel therapeutic agents for improving bone health is important. The causal metabolic pathway identified in this study may provide insight on therapeutic agent development. Moreover, the associated metabolites may be used as a biomarker of osteoporosis or potentially as a dietary supplement to improve BMD. Follow‐up studies are now underway to evaluate the importance of these findings.

In conclusion, circulating metabolites measured using a high‐throughput metabolomic technique explained more than one‐third of the variance of BMD in a large female population. Fifteen unique metabolites showed direct association with BMD variation, four of which were found to be causally associated using genetic instruments. The direct association between these metabolites and BMD were replicated in an independent Chinese population. Combining both genomic and metabolomic data provided a unique platform for elucidating the role of new pathways in the pathogenesis and diagnosis of osteoporosis.

## Disclosures

RPM is an employee of Metabolon, Inc. All other authors state that they have no conflicts of interest.

## Supporting information

Supporting Data S1.
